# Visualizing the Translocation and Localization of Bacterial Type III Effector Proteins by Using a Genetically Encoded Reporter System

**DOI:** 10.1128/AEM.03418-15

**Published:** 2016-04-18

**Authors:** Jayde A. Gawthorne, Laurent Audry, Claire McQuitty, Paul Dean, John M. Christie, Jost Enninga, Andrew J. Roe

**Affiliations:** a Institute of Infection, Immunity, and Inflammation, University of Glasgow, Glasgow, United Kingdom; b Institut Pasteur, Paris, France; c School of Biomedical Sciences, Newcastle University, Newcastle, United Kingdom; d Institute of Molecular, Cell, and Systems Biology, University of Glasgow, Glasgow, United Kingdom; Washington State University

## Abstract

Bacterial type III secretion system (T3SS) effector proteins are critical determinants of infection for many animal and plant pathogens. However, monitoring of the translocation and delivery of these important virulence determinants has proved to be technically challenging. Here, we used a genetically engineered LOV (light-oxygen-voltage) sensing domain derivative to monitor the expression, translocation, and localization of bacterial T3SS effectors. We found the Escherichia coli O157:H7 bacterial effector fusion Tir-LOV was functional following its translocation and localized to the host cell membrane in discrete foci, demonstrating that LOV-based reporters can be used to visualize the effector translocation with minimal manipulation and interference. Further evidence for the versatility of the reporter was demonstrated by fusing LOV to the C terminus of the Shigella flexneri effector IpaB. IpaB-LOV localized preferentially at bacterial poles before translocation. We observed the rapid translocation of IpaB-LOV in a T3SS-dependent manner into host cells, where it localized at the bacterial entry site within membrane ruffles.

## INTRODUCTION

Imaging the complex dynamics of bacterium-host cell interactions using light microscopy is a key step in understanding bacterial pathogenesis and identifying new possibilities to interfere with the infection process. Central to the pathogenesis of many Gram-negative bacteria is the type III secretion system (T3SS), an organelle that facilitates the injection of effector proteins from the bacterial cytoplasm into host cells. Effectors of pathogenic Escherichia coli, Shigella, Salmonella, and Yersinia perform very diverse functions, including regulation of actin dynamics to facilitate their own attachment or invasion, subversion of endocytic trafficking, blocking of phagocytosis, modulation of apoptotic pathways, and manipulation of innate immunity, as well as host responses (1, 2).

The use of innovative imaging assays and probes to study the cellular microbiology of effector proteins is becoming commonplace as limitations in image capture, data processing, and suitable probes are overcome. Current light microscopy methods to study effectors include fluorescence resonance energy transfer (FRET), fluorescent detection after secretion, direct fluorescence labeling, and recombinant reporter assays following translocation (1). Each method is designed to allow effectors to be analyzed at specific points such as the time of injection, identification of interacting host proteins, or final localization within host cells.

A FRET reporter for T3SS effector translocation has been used in numerous studies such as one by Auerbach et al., wherein the subset of immune cells targeted by injected Yersinia pestis effectors during infection was determined (3). Mills et al. also used the system to determine the timing and hierarchy of effector translocation in enteropathogenic E. coli (EPEC) (4). For the study of effector proteins, a commonly adopted approach involves fusing the effector to a β-lactamase reporter (5). The host cells are labeled with the nonfluorescent CCF2/AM substrate that is rapidly converted by cellular esterases to fluorescent CCF2. Excitation of the coumarin moiety results in FRET to a fluorescein moiety that emits a green fluorescence signal. Translocation of an effector fused to TEM-1 induces catalytic cleavage of the CCF2 β-lactam ring, affecting the FRET. This produces a detectable and measurable change in CCF2 fluorescence from green to blue emission.

Fluorescent reporters that are detected after translocation have also been used successfully to monitor the timing of effector delivery into host cells. The use of a split-green fluorescent protein (GFP) system (6) overcomes the limitation of the type III secretion system (T3SS) to secrete partially folded polypeptides by fusing part of the GFP fluorophore to the effector and expressing the remaining component in the host cell. Upon successful translocation, the two halves are united to form a functional molecule suitable for immunofluorescence, as demonstrated for Salmonella SPI2 effectors (6). The use of such a system may also provide spatial information as the final localization can be determined, a marked advantage compared to the FRET-based system described above.

Direct labeling of the effector has been achieved with a tetracycline motif tag (4Cys) fused to the Shigella effectors IpaB and IpaC. The effectors are then detected using a fluorescein-based biarsenic dye (FlAsH) that becomes fluorescent upon binding to the 4Cys motive. FlAsH was successfully used to monitor Salmonella Typhimurium effector translocation in real time. However, detection dyes are often toxic to eukaryotic cells, such as the biarsenic dye in FlAsH (7) and CCF4 in FRET (5). Moreover, until now, no reporter has allowed the generation of a fusion protein to detect expression of the effector inside bacteria and its subsequent translocation into host cells in real time.

We have previously used derivatives of the LOV domain to monitor protein expression and purification (8). LOV domains are light-sensing motifs found in diverse photoreceptor proteins from bacteria, fungi, and plants (9). LOV domains bind the chromophore flavin mononucleotide (FMN) and emit green fluorescence when irradiated with blue/UV light. Advantages of LOV domains include their small size (∼10 kDa) combined with an innate ability to acquire their flavin fluorophore from the cellular environment. Using this approach we previously showed that the LOV-domain variant iLOV is effective as a fluorescent reporter of protein production from pET-based vectors (10). Addition of iLOV did not impede functionality of the effector protein EspG upon microinjection into normal rat kidney (NRK) cells, leading to disruption of the Golgi apparatus, as had previously been observed for EspG (11). However, EspG-iLOV fluorescence in NRK cells could not be observed possibly due to its disperse localization and/or a detection limitation of iLOV fluorescence within eukaryotic cells.

Here, we sought to improve iLOV and determine whether effectors could be monitored during secretion through the T3SS apparatus. To this end, we initially focused on the translocated intimin receptor (Tir) and the mitochondrial associated protein (Map). The observation that enterohemorrhagic E. coli (EHEC) translocates its own receptor into mammalian cells was described more than a decade ago (12). Tir inserts into the plasma membrane in a hairpin-loop conformation consisting of an extracellular domain flanked by two transmembrane and N- and C-terminal cytoplasmic domains (13). The extracellular domain of Tir binds Intimin on the bacterial cell surface, triggering multimerization, and clustering of Tir beneath attached bacteria (14). Elegant studies have demonstrated that EHEC Tir then binds a second effector, EspFu (also termed TccP) triggering the activation of N-WASP-Arp2/3-mediated actin assembly (15, 16). This activation initiates downstream signaling events, leading to the formation of the characteristic actin-rich pedestals.

Map, named according to its cellular target, is also a well-characterized effector. Map has been demonstrated to possess three distinct and independent functions. First, it interferes with the cellular ability to maintain mitochondrial membrane potential, triggering mitochondrial swelling and damage (17). Second, in initial stages of EHEC infection, Map is responsible for the transient formation of filopodium-like structures at the sites of bacterial infection (18). Third, Map is essential for disruption of intestinal barrier function and alteration of tight junctions, an activity that is independent of mitochondrial targeting (19).

Given that Tir and Map functionality are well understood, we felt these effectors provided ideal candidates to evaluate whether LOV can be used to monitor T3SS translocation. Furthermore, we evaluated the ability of LOV to track T3SS effector trafficking in another bacterium, namely, Shigella flexneri. We focused on the translocator/effector IpaB. This protein is produced and stored intrabacterially before the contact with host cells that induces the T3SS (20). Furthermore, it forms a complex with IpaD at the tip of the T3SS needle. It has previously been shown that prestored IpaB is entirely released from the bacteria during host cell contact (7). IpaB acts as a translocator, forming pores for the translocation of other effector proteins. Within the host cells, IpaB has been suggested to be involved in the activation of caspase-1 in macrophages and modulation of the cell cycle progression in epithelial progenitor cells. Like Tir, IpaB is therefore well characterized and provided an excellent model protein to test whether LOV can be used to track effectors. We found that both Tir and IpaB could be tagged with LOV and were successfully translocated into host cells. Overall, our work shows that LOV domains can be used as simple, genetically encoded reporters to monitor effector protein expression and translocation.

## MATERIALS AND METHODS

### Generation of an E. coli O157:H7 strain expressing red fluorescent protein (RFP).

Gene synthesis (DNA 2.0) was used to generate plasmid pJ241-RFP, containing a gene encoding an enhanced RFP under the control of a pTAC promoter. The primers RfpFor (5′-GTGTCGCCCTTATTCGACTCTAT-3′) and RfpRev (5′-CGCCCTTATTCGACTCACTATAGAAGTTCC-3′) were used to amplify the RFP gene and the pTAC promoter. The product was purified using a Qiagen PCR purification kit, digested with BamHI, repurified, and then ligated using standard conditions into the BamHI site of pAJR26 (21). Allelic exchange was performed as previously described (22) using ZAP1193 (21), a derivative of strain NCTC12900, as the recipient strain.

### Creation of effector phiLOV expression plasmids.

DNA 2.0 was used to synthesize plasmid, pJAG03 that facilitates in-frame cloning of effectors and promoters with a variant of LOV, termed phiLOV. The primers RfpFor and ptacNdeIKpnI (5′-CTTCACCGGTACCAACCATATGTTATCCTCC-3′) were used to amplify the pTAC promoter; this was then ligated into pJAG03 to create pJAG07. Tir and its native promoter was digested from pAJR133, gel purified, and ligated into pJAG03 to create the plasmid pJAG13. The region encompassing the promoter and open reading frame encoding the EHEC effector Map was amplified using primers (5′-CGAGATCTGCACACTCCAGTATCCATTCA-3′ and 5′-CGGGTACCCAATCGGGTATCCTGTACATG-3′) from TUV93-0. The resulting product was cloned into pJAG13 in place of Tir to create pCMQ1. To create pBADIpaB-phiLOV, phiLOV was first amplified from pJAG13 using the primers XbaLOV (5′-CGTAGCTCTAGAATGATCGAGAAGAGCTTTG-3′) and HindLOV (5′-CGTAGCAAGCTTTTAACGTGGTCGGAACCA-3′). IpaB was amplified using the primers NdeIpa (5′-CGTAGCGCTAGCATGCATAATGTAAGCACCAC-3′) and XbaIpaB (5′-CGTAGCTCTAGATCAAGCAGTAGTTTGTTGCAAA-3′) to eliminate the native stop codon and allow creation of the in-frame fusion. The PCR products were cloned successively into pBAD18, creating pBADIpaB-phiLOV, and checked by sequencing.

### Measurement of relative fluorescence.

ZAP193Δ*escN*, a derivative of NCTC12900 that is unable to secrete effectors, was transformed with pJAG13 (pTir-phiLOV), pCMQ1 (pMap-phiLOV), or pAJR75 (pTir-GFP). Bacteria were cultured overnight in Luria-Bertani medium, subcultured into minimal essential medium (MEM) containing 50 mM HEPES in a 96-well plate to an optical density (OD) of 0.1, and grown at 37°C and 200 rpm, with readings taken at 600 and 488 nm every 30 min until an OD of 0.8 was reached. The assay was performed in triplicate and mean fluorescence readings corrected against a wild-type (WT) strain with no plasmid.

### Preparation of secreted proteins.

Bacteria were cultured in 50 ml of MEM-HEPES at 37°C and 200 rpm to an OD at 600 nm (OD_600_) of 0.8. The bacterial cells were pelleted by centrifugation at 4,000 × *g* for 10 min, and the supernatant was separated. The proteins were precipitated overnight with 10% trichloroacetic acid (TCA), and separated by centrifugation at 4,000 × *g* for 30 min at 4°C. The proteins were suspended in 150 μl of 1.5 M Tris-HCl (pH 8.8).

### Immunoblotting.

Proteins were separated by SDS-PAGE according to standard methods, and Western blotting was performed as previously described with α-Tir (a gift from Trinad Chakraborty), α-Sigma-70 (Neoclone), α-LOV (8), and α-calnexin antibodies (Pierce). For the secreted proteins, cell culture supernatants were syringe filtered (0.45-μm pore size) and precipitated with 10% (vol/vol) TCA (Sigma) overnight at 4°C. Secreted proteins were harvested by centrifugation at 4,000 rpm (4°C) for 1 h. Protein pellets were resuspended in Tris-HCl (pH 8.0), and equivalent volumes were analyzed by SDS-PAGE. For the whole-cell fractions, 20 μg of total protein were loaded and probed. Experiments were repeated a minimum of three times.

### Bacterium-cell adhesion assay.

Bovine embryonic lung (EBL) and HeLa cells were seeded onto coverslips in a 24-well plate with ca. 60% confluence and incubated overnight at 37°C with 5% CO_2_. EHEC bacteria were cultured in 50 ml of MEM supplemented with 50 mM HEPES and antibiotics as required. The cultures were incubated at 37°C and 200 rpm until an OD_600_ of 0.6 was reached. The bacteria were diluted with warm MEM-HEPES and added to the cells at an approximate multiplicity of infection of 30. The 24-well plate was centrifuged at 400 × *g* to initiate bacterial contact with the eukaryotic cells. The plate was then incubated at 37°C with 5% CO_2_. For the induction of the S. flexneri T3SS, bacteria were cultured in tryptic soy broth containing chloramphenicol and Congo red.

### Host cell membrane purification.

Bacterial cell adhesion assays were performed as described above. After 4 h of EHEC adhesion to EBLs, the cells were treated with 50 μg of gentamicin/ml at 37°C for 30 min to remove all bacteria. The host cells were then trypsinized and washed, and the host cell components were separated by using a Mem-PER eukaryotic membrane protein extraction reagent kit (Pierce), as described by the manufacturer.

### Real-time bacterium-cell adhesion assay.

HeLa cells were seeded into 96-well Nunc plates with ca. 60% confluence and incubated overnight at 37°C with 5% CO_2_. The following day the cells were labeled with CellTrace-DDAO to prepare them for imaging. Bacteria were cultured in 10 ml of MEM without phenol red (MEM-no phenol red; Sigma) supplemented with 50 mM HEPES and antibiotics as required. The cultures were incubated at 37°C and 200 rpm until an OD_600_ of 0.6 was reached. The bacteria were diluted with warm MEM, 50 mM HEPES, and added to cells at an approximate multiplicity of infection of 20. The 96-well plate was centrifuged at 400 × *g* to initiate bacterial contact with the HeLa cells. The plate was washed every 60 min to prevent overgrowth of unattached bacteria in media. The expression and translocation of individual bacteria was monitored (see Fig. 4e for a single example). Some 15 bacteria were measured in this manner; however, they attach to host cells at different rates, resulting in the population being nonsynchronous. As a technical point, we found that HeLa cells were more suitable for fluorescence imaging over extended periods since they were better able to tolerate repeated exposure to ultraviolet light compared to the EBLs. HeLa cells have been used successfully to study pedestal formation of both EPEC and EHEC (23).

### Fluorescence imaging.

Fluorescence imaging, such as testing time points for optimum expression and translocation, was performed using a Zeiss AxioImager M1 widefield fluorescence microscope equipped with a Hamamatsu Orca CCD camera and appropriate fluorescence filter sets. Imaging of the precise localization of the Tir-phiLOV fusion during the translocation process, such as those shown in Fig. 3d to h and Fig. 4a to d, were obtained using a DeltaVision RT epifluorescence imaging system (Applied Precision) and SoftWoRx software. Rapid three-dimensional time-lapse imaging of Tir-phiLOV (Fig. 4e to h) and IpaB-phiLOV (Fig. 5) were obtained using a spinning disk confocal microscope using the 488-nm laser for phiLOV excitation (Perkin-Elmer). Data were captured and analyzed using Volocity Suite software (Perkin-Elmer), allowing quantification of two-dimensional (2D) images (pixels) or 3D images (voxels).

## RESULTS

phiLOV2.1, a derivative of the iLOV domain was used throughout the study given its enhanced photostability and fluorescence (24). Gene synthesis (DNA 2.0) was used to codon optimize phiLOV2.1 to facilitate expression in E. coli and S. flexneri. The resultant domain, henceforth referred to as phiLOV, was cloned into pACYC create pJAG03. The promoter region and coding sequence for the EHEC effector proteins Tir and Map were cloned from strain TUV93-0 into the pJAG03 backbone to create pJAG13 and pCMQ1, respectively, as described in Materials and Methods. To allow bacterial imaging without the need for antibody staining, the gene encoding a red fluorescent protein (RFP) optimized for bacterial expression was stably integrated into the EHEC genome in place of *lacZ*.

**FIG 1:**
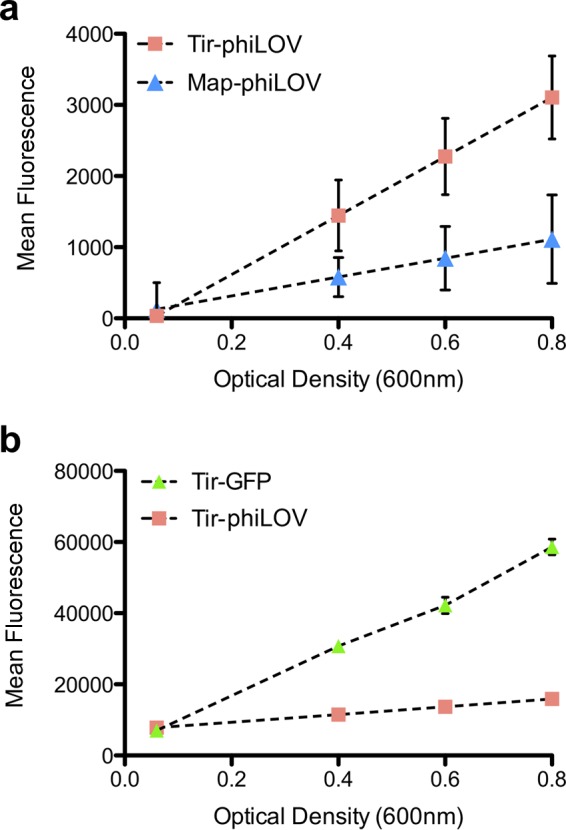
Analysis of relative fluorescence of EHEC effector-phiLOV fusion proteins under T3SS-inducing conditions. (a) EHEC expressing either Tir-phiLOV or Map-phiLOV was grown under T3SS-inducing conditions to determine whether expression of each effector could be measured using a simple plate reader assay. Each effector-phiLOV fusion was measured in triplicate, and the mean fluorescence was monitored over exponential growth. (b) Fluorescence of EHEC expressing either Tir-GFP or Tir-phiLOV was monitored to determine how the Tir-phiLOV fusion would perform directly compared to Tir-GFP.

To explore the properties of Tir-phiLOV and Map-phiLOV, we evaluated their relative fluorescence when expressed in EHEC under the control of their native promoters. Since it was possible that phiLOV fusions might be secreted, expression studies were performed in an EHEC T3SS-deficient strain that lacks the ATPase (EscN) ensuring that all the fusion protein was retained in the bacterial cytoplasm. This allowed for a more direct comparison between different reporters and constructs without the possibility of protein secretion. Bacteria were cultured in MEM-HEPES media to induce the expression of the T3SS and the level of fluorescence for both phiLOV reporters monitored throughout the growth phase. Fluorescence readings showed that Tir-phiLOV was approximately three times brighter than Map-phiLOV (Fig. 1a). Indeed, we concluded that the expression of Map-phiLOV was too low to warrant further study, a limitation we discuss further below. To determine the relative expression of Tir-phiLOV compared to existing GFP reporters, we also compared the Tir-phiLOV reporter with a Tir-GFP reporter (pAJR75) (25). Both plasmids comprised the same promoter sequence and plasmid backbone, ensuring that the only variable was the fluorescent reporter being tested. The GFP reporter displayed fluorescence that accumulated over time. In comparison, the phiLOV reporter was less fluorescent, around 2.5 to 3 times less than the GFP through the entire exponential phase (Fig. 1b). However, despite being less fluorescent compared to GFP, the Tir-phiLOV reporter was detectable on a simple fluorescence plate reader at similar growth stages to Tir-GFP, suggesting that it would be readily imaged within single cells.

**FIG 2:**
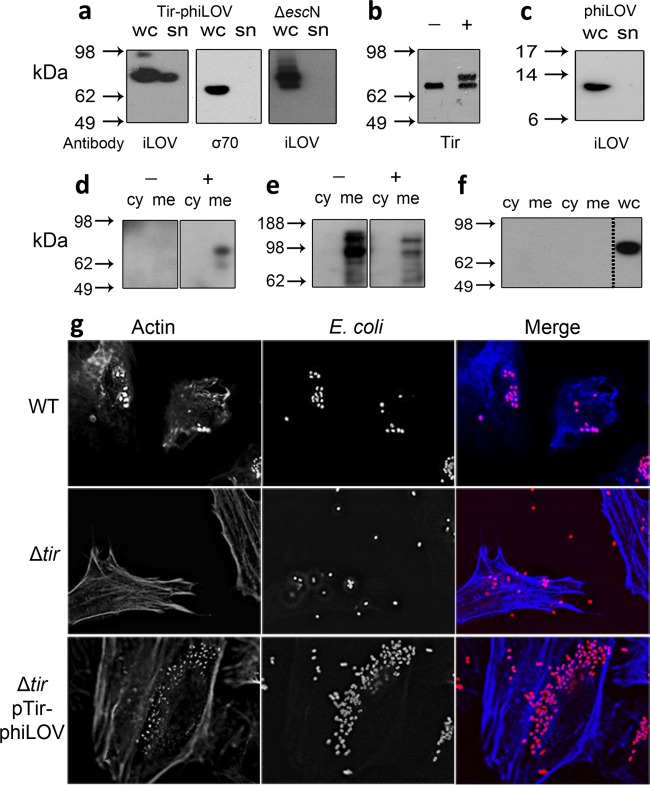
Analysis of Tir-phiLOV secretion and translocation. EHEC strains were cultured in T3SS-inducing conditions and harvested at an OD_600_ of 0.6. Samples were centrifuged to yield the supernatant fraction (sn), and the bacterial pellet was lysed with Bugbuster (whole cell [wc]). (a) EHEC and EHECΔ*escN* transformed with a pTir-phiLOV probed with α-iLOV antibodies. An α-σ^70^ immunoblot acted as a control for bacterial lysis. (b) Comparison of Tir-phiLOV and Tir secretion from EHEC transformed with empty vector (−) or pJAG13 (+) after probing with an α-Tir antibody. (c) EHEC transformed with a pTAC-phiLOV plasmid probed with α-iLOV antibodies shows no secretion into the supernatant. To test translocation, a bacterium-host cell adhesion assay was performed using EHEC with either no plasmid (−) or pTir-phiLOV (+) on EBL eukaryotic cells. Four hours after addition of the bacteria, the EBL cells were treated with gentamicin, harvested, and fractionated into cytoplasmic (cy), membranes (me), and lysed bacteria (wc). The fractions were probed with α-iLOV antibodies (d), α-calnexin antibodies (e), and α-σ^70^ antibodies (f), with the lysed bacteria acting as a positive control (wc). (g) To evaluate whether Tir-phiLOV was functional, WT EHEC, a Δ*tir* mutant, and the Δ*tir*/Tir-phiLOV strain were added to HeLa cells and fixed at various time points after the infection. Condensation of host cell actin was visualized by use of Alexa Fluor-labeled phalloidin. Bacteria were detected by addition of α-O157 antibodies. Deletion of Tir reduces the ability of bacteria to attach to host cells and prevents condensation of host cell actin, a trait that was restored by transformation with the plasmid expression Tir-phiLOV.

To determine whether the Tir-phiLOV fusion could indeed be secreted via the T3SS, wild-type EHEC was transformed with pJAG13 and cultured in a MEM-HEPES media that induces expression of the T3SS. Immunoblotting showed that the Tir-phiLOV fusion protein was detectible in both whole-cell and secreted fractions (Fig. 2a). Monitoring the levels of σ^70^, a bacterial cytoplasmic protein, confirmed that Tir-phiLOV in the secreted fraction did not result from bacterial cell lysis (Fig. 2a). Moreover, deletion of EscN (Δ*escN*), the ATPase required for T3SS activity, prevented export of the Tir-phiLOV fusion protein, allowing detection only in the whole-cell fraction (Fig. 2a). To compare the secretion of native Tir and Tir-phiLOV, the secreted protein fractions from WT EHEC and the WT transformed with pAJG13 were probed using antibodies for Tir. This revealed both native Tir and Tir-phiLOV in the supernatant of the transformed strain in equal amounts (Fig. 2b). However, when phiLOV was expressed independently of Tir, phiLOV was only detected in bacterial whole-cell fractions, indicating that the fluorescent tag itself is not secreted independently of the effector (Fig. 2c). These data supported the notion that Tir-phiLOV could be secreted via the T3SS, a key advantage of the phiLOV reporter system over GFP.

The next question was to address whether Tir-phiLOV could be translocated into eukaryotic cells. This was examined using a bacterium-host cell adhesion assay. Bacteria expressing Tir-phiLOV were used to challenge embryonic bovine lung (EBL) epithelial cells for 240 min. Biochemical fractionation showed that Tir-phiLOV was present in the eukaryotic membrane (Fig. 2d), demonstrating that phiLOV could be translocated into host cells. Eukaryotic membrane purity and the effective removal of the bacteria were verified by immunoblotting for the integral membrane protein α-calnexin (Fig. 2e) and the bacterial cytoplasmic protein σ^70^, respectively (Fig. 2f).

Given that Tir-phiLOV was effectively secreted by the T3SS and translocated into host cell membranes, the next key question was to determine whether it was functional. To address this question, we performed cell infection assays using WT EHEC, the Δ*tir* mutant, and the Δ*tir* mutant expressing Tir-phiLOV. WT bacteria could be seen to adhere intimately to the host cells, and the formation of distinct pedestals could be seen after staining using fluorescein isothiocyanate-phalloidin (Fig. 2g). This phenotype is due to the accumulation of host cell actin after recruitment by Tir at the site of bacterial attachment. Deletion of *tir* resulted in the total absence of actin pedestals (Fig. 2g), a phenotype that could be effectively rescued by Tir-phiLOV (Fig. 2g), showing that Tir-phiLOV was both translocated and functional.

**FIG 3:**
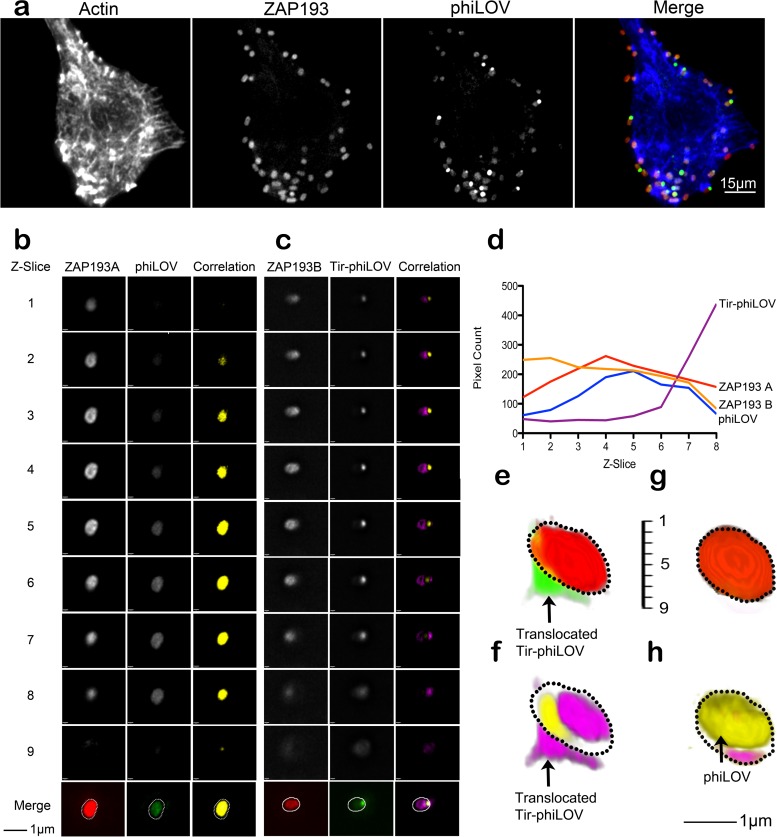
Imaging of Tir-phiLOV translocation. EHEC strains were transformed with pTAC-phiLOV or Tir-phiLOV and added to EBL eukaryotic cells. After 2 h, the bacteria were fixed, and images were obtained and quantified. (a) WT EHEC transformed with Tir-phiLOV showed expression of the reporter after cell contact. The bacterial cytoplasm was marked using the chromosomal RFP reporter and host cell actin stained using labeled phalloidin. (b and c) Expression and localization of phiLOV (b) and Tir-phiLOV (c) of bacteria attached to host cells. The images show Z-slices from the “top” (slice 1) to the “bottom” (slice 9) of attached bacteria. Areas of correlation between phiLOV and RFP are colored yellow, whereas pink shows areas where no correlation was measured. (d) Quantification of the number of pixels associated with each Z-slice for the Tir-phiLOV, phiLOV, and bacterial RFP cytoplasmic channels (ZAP193A and -B). (e to h) 3D, false-colored projections of the bacteria show that Tir-phiLOV (green) is spatially distinct from the bacterial cytoplasm (red). Areas of correlation between phiLOV and RFP are highlighted using yellow with pink, showing areas where no correlation was measured. Representative images are shown; a minimum of 10 individual bacteria were analyzed per experiment.

Having established that Tir-phiLOV is secreted, translocated, and indeed functional, fluorescence microscopy was used to visualize the expression of Tir-phiLOV within individual bacteria. EBL cells were infected with EHEC expressing Tir-phiLOV. After fixation, Tir-phiLOV expression could be seen in the vast majority (>90%, *n* = 300) of EHEC bound to the EBL cells (Fig. 3a) approximately 240 min after the initial infection. The level of Tir-phiLOV expression varied considerably, a trait consistent with the known heterogeneity associated with expression of LEE5 (25). In order to ascertain whether Tir-phiLOV could be visualized after its export, successive Z-slides were analyzed and compared to the bacterial cytoplasmic channel, visualized using RFP. As a control, phiLOV expressed independently of Tir was found to colocalize very closely to the cytoplasmic RFP signal, demonstrating that in the absence of an effector fusion phiLOV shows no distinct localization pattern (Fig. 3b). In contrast, comparison of Tir-phiLOV and the cytoplasmic channels showed a distinct localization, suggesting that the fusion protein was localized adjacent to a proportion of the bacteria (Fig. 3c). Quantification of the pixel density associated with each channel demonstrated a clear difference in the distribution of Tir-phiLOV compared to phiLOV alone (Fig. 3d). Analysis of successive Z-slices demonstrated that Tir-phiLOV was indeed highly localized within individual bacteria and that a high proportion of the fusion protein was localized directly adjacent to the bacterial cell (Fig. 3e and f), again in stark contrast to expression of the phi-LOV domain alone, which showed excellent colocalization with the bacterial cytoplasm (Fig. 3g and h).

**FIG 4:**
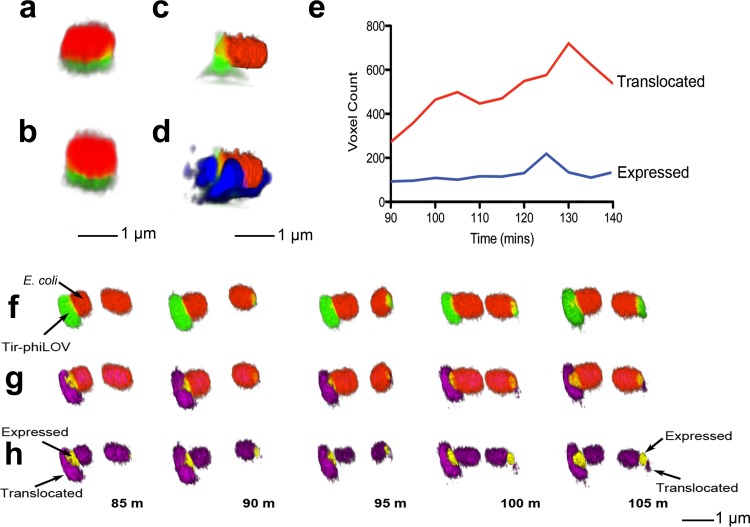
Imaging of Tir-phiLOV colocalization with host cell actin and translocation in real-time. EHEC were transformed with Tir-phiLOV and added to HeLa cells. (a and b) 3D, false-colored projections of the attached bacteria to show bacteria with multiple Tir-phiLOV foci. (c and d) 3D, false-colored projections of the attached bacteria to show association of Tir-phiLOV with host cell actin (false-colored blue in panel d). (e) Real-time monitoring of Tir-phiLOV expression and translocation from a single bacterium. (f to h) 3D false-colored projections of a 20-min time course of EHEC attachment and Tir-phiLOV expression on HeLa cells. (f) EHEC (red) and Tir-phiLOV (green). (g) EHEC (red) with positive (yellow; overlap with green channel) and negative (purple; no overlap with green channel) correlation channels. (h) The positive (yellow) and negative (purple) correlation of the red channel (EHEC) and green (Tir-phiLOV). Note the negative correlation channel beside the area corresponding to the bacteria, indicating translocated Tir-phiLOV, and the positive channel indicates the overlap of the red and green channels, indicating Tir-phiLOV still inside the bacteria.

The majority of bacteria (60% at 60 min postinfection, *n* = 60) were found to translocate Tir-phiLOV at a single well-defined focal point, whereas a smaller proportion (30%, *n* = 30) showed two or, in some cases, several (10%, *n* = 25) clearly defined areas of Tir-phiLOV translocation, (Fig. 4a and b; see also Movie S1 in the supplemental material), highlighting that effector trafficking that may be highly targeted to selected T3SS, and this subsequently results in discrete translocation points into the host cell. Additional staining showed partial colocalization with host cell actin, confirming our previous finding that Tir-phiLOV was both translocated (Fig. 4c) and capable of mediating actin polymerization (Fig. 4d). One caveat is that, since WT bacteria were used, the variation in the number of foci observed may result from competition between native Tir and Tir-phiLOV for export by the T3SS.

Having established an optimum time frame for Tir-phiLOV translocation, visualizing the localization of this effector was attempted in real time. The real-time imaging was challenging, mainly due to the extended period required when monitoring the infection process, taking hours rather than a few minutes. This resulted in movement of the host cells and the attached bacteria, making their imaging quite problematic. Moreover, phototoxicity and photobleaching also become significant issues during repeated imaging. However, we did succeed in tracking bacteria for a 50-min period after addition to host cells. Using spinning-disk confocal microscopy, it was possible to measure both the cytoplasmic expression and translocation of Tir-phiLOV by individual bacteria during the host cell attachment process (Fig. 4e). This imaging also highlighted the heterogeneity of Tir-phiLOV expression with adjacent bacteria showing different timing of Tir-phiLOV expression (Fig. 4f to h).

**FIG 5:**
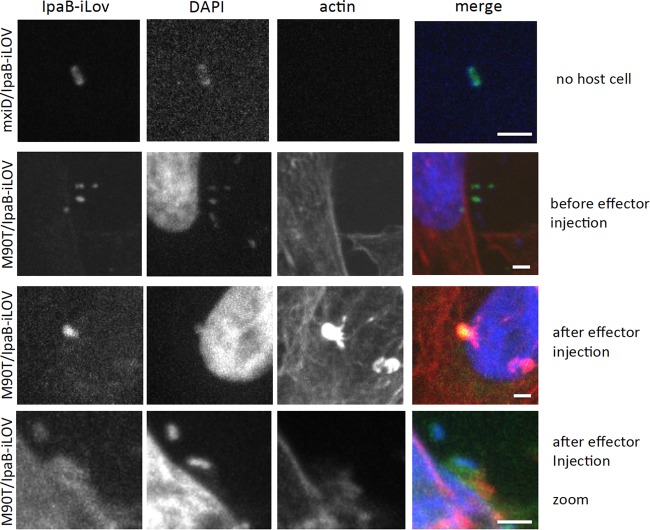
Translocation of IpaB-LOV in Shigella flexneri. WT S. flexneri and an isogenic T3SS-deficient strain (Δ*mxiD*) were transformed with the IpaB-phiLOV construct. Bacterial DNA and host nuclei were stained with DAPI (blue), and actin focus formation was tracked by using phalloidin-rhodamine (red). Before host cell contact, IpaB-phiLOV localizes to the bacterial poles (upper panel and second panel). After translocation, it is located at the forming entry foci in the vicinity of the bacterium (lower two panels).

**FIG 6:**
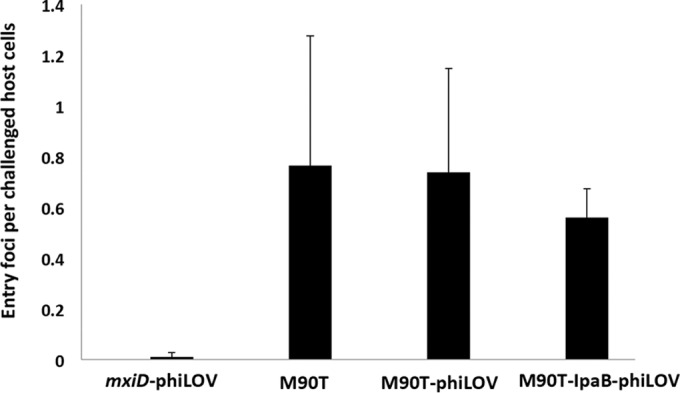
Quantification of bacterial uptake in S. flexneri. Bacterial invasiveness for the shown strains was quantified scoring Shigella-induced actin rearrangements within host cells at the bacterial entry site using phalloidin-rhodamine. Expression of IpaB-phiLOV or phiLOV alone did not affect the internalization of Shigella after 45 min of host cell challenge. For each condition, 100 cells were scored (*n* = 3). Error bars indicate the standard deviations.

The simplicity of the phiLOV reporter makes it an attractive tool that might be applied to study translocation events in a wide range of T3SS-expressing bacteria. We wanted to test this potential wider applicability using a completely different pathogen and effector combination. To this end, we tested Shigella T3SS effector translocation with an IpaB-phiLOV fusion. We chose IpaB because its secretion kinetics have been analyzed previously (7), it can be modified at its C terminus without significantly impeding its secretion, and its function has been analyzed in some detail (see the introduction). It is expressed at high levels compared to other effectors; therefore, it is a good candidate for the establishment and validation of a new effector labeling approach. The fusion protein was generated by cloning the sequences encoding *ipaB* and phiLOV into pBAD18, thereby creating pBADIpaBphiLOV, allowing inducible expression upon the addition of arabinose (see Materials and Methods). An advantage of Shigella is that interactions with host cells result in a rapid injection of T3SS effectors and effects on the host cell in comparison to EHEC. Shigella effector translocation occurs within minutes rather than hours after initial infection (7). When expressed in the WT S. flexneri strain M90T, IpaB-phiLOV could be readily visualized within the bacteria, yielding a typical polar localization that has been previously identified for a number of bacterial effectors using different fluorescent techniques (7, 26) (Fig. 5). Remarkably, after a time course between 15 and 45 min of contact with the epithelial host cell line HeLa, fluorescence was rapidly dissipated from the bacterial cytoplasm, a finding consistent with its translocation. On the other hand, we could detect IpaB-phiLOV during the investigated time course within the targeted HeLa cells in proximity to the invading bacteria. IpaB-phiLOV localization within host cells was similar to the localization of IpaB using other fluorescent techniques (7) highlighting the potential of the phiLOV labeling only minimally perturbing the localization of the tagged effectors. As an additional control, an isogenic Δ*mxiD* strain of S. flexneri was also used. In this T3SS-deficient mutant, IpaB-phiLOV was retained within the bacterial cell, and no attachment to host cells was observed (Fig. 5). Quantification of the level of fluorescence within the bacteria to that outside revealed the dramatic loss of the effector pool within the injecting bacteria. We also tested whether Shigella invasion of HeLa cells was perturbed through the expression of IpaB-phiLOV. Scoring the number of entry foci as a marker for ongoing bacterial uptake for M90T, M90T/IpaB-phiLOV, M90T/phiLOV, and the noninvasive mutant mxiD/IpaB-phiLOV, we could not detect a significant difference in the number of entry foci for the WT strains expressing the different fluorescently tagged proteins (Fig. 6). This showed that IpaB-phiLOV expression did not perturb the entry of the pathogen into the host cells.

## DISCUSSION

Previous studies have used a variety of technologies to monitor effector protein translocation, including β-lactamase, FlAsH/tetracysteine, and split-GFP approaches that require the two “halves” of the protein to reassociate and mature for reporter activity (6). Each approach has its inherent strengths and weaknesses that have been reviewed extensively (1). The principal advantages of phiLOV as a reporter are its intrinsic and oxygen-independent fluorescence (27) combined with its small size. We also note inherent limitations. The Tir-phiLOV fusion was less fluorescent than the equivalent GFP reporter, so for applications such as simple transcriptional readout, with no requirement for secretion, GFP still provides the benchmark. Indeed, previous work has directly compared the quantum yields (QY) of LOV-based reporters and shown them to be significantly lower (QY = 0.2 to 0.4) to that of GFP (QY = 0.6) (28), which is consistent with our data.

Moreover, the native Tir promoter is strong, providing an ample level of Tir-phiLOV that could be detected in both the bacterium and the host membrane. In contrast, the expression of Map was found to be too low to warrant detailed study using our experimental setup. Indeed, effector proteins with poorly defined or weak native promoters might benefit from using the arabinose-inducible plasmid that we used for the study of IpaB. The dramatic loss of intrabacterially stored IpaB that we observed was previously detected using the 4Cys-FlAsH labeling approach (7). This points at the efficiency of the Shigella T3SS that is capable to translocate large amounts of proteins within short periods of time. We expressed IpaB in the present study and in the previous study exogenously using a pBAD plasmid. It remains to be determined how the activation of the T3SS affects the protein expression of IpaB. Despite this, our data suggest that the rate of secretion is much higher than the replenishment within the bacterium. It is also worth noting that translocation of IpaB could be measured within 15 min, but not in real time, a trait we feel is likely to be due to a requirement for maturation of the phiLOV effector fusion. However, the inherent simplicity of being able to tag an effector with a genetically encoded reporter and monitor the entire expression and translocation process is clearly of huge benefit to researchers. Tracking effectors using LOV derivatives also presents the opportunity to perform correlative light electron microscopy (CLEM), providing a further increase in resolution and insights into effector functionality (29).

Collectively, the data presented here demonstrate the utility of the phiLOV-based reporter system to effectively monitor T3SS dynamics and effector protein trafficking at high resolution in a spatial-temporal manner. When fused to Tir, phiLOV was translocated through the T3SS and inserted into the host eukaryotic membrane. Crucially, in a Δ*tir* strain, the expression of Tir-phiLOV rescued the ability of the bacteria to form attaching-and-effacing lesions upon host cell contact. This demonstrates that Tir-phiLOV is functional upon its translocation. We previously showed that EspG was also functional when tagged with iLOV, a less fluorescent predecessor of phiLOV. Moreover, using an entirely different pathogen and effector combination, we showed that IpaB from S. flexneri could also be monitored using phiLOV.

Further studies could couple reporter fusions for basal apparatus proteins and phiLOV-tagged effectors to elegantly address some of the questions raised by this study. For example, it would be fascinating to dissect whether bacteria activate T3SS that are in contact with host cells. Moreover, there is great potential for applying phiLOV to the study of other effectors to obtain a spatiotemporal “map” of their subcellular localization during the infection process. This is particularly relevant since certain effectors target multiple sites, for example, EspF, a multifunctional effector with more than five proven eukaryotic targets (30). However, we acknowledge that the reporter may not be suitable for all effectors. Effectors with a low level of translocation would invariably be much harder to detect and may require strong inducible reporters to maximize their production. It would also be interesting to evaluate whether addition of the phiLOV fusion alters effector stability in host cells. Elegant studies have shown that effectors can have antagonistic functions and that degradation by host cell proteolysis is central to their interplay (31). Again, the use of inducible promoters might provide an effective way of controlling translocation and allowing this interesting aspect of effector biology to be further dissected.

## Supplementary Material

Supplemental material
