# Supplementary material for: Visualizing the Translocation and Localization of Bacterial Type III Effector Proteins by Using a Genetically Encoded Reporter System
Source: Appl Environ Microbiol. 2016 Apr 18;82(9):2700–8. doi: 10.1128/AEM.03418-15 (PMC4836418; doi:10.1128/AEM.03418-15)
Supplement: Supplemental material [file supp_82_9_2700__index.html]

Supplemental material 

# Visualizing the Translocation and Localization of Bacterial Type III Effector Proteins by Using a Genetically Encoded Reporter System

## Supplemental material

- Supplemental file 1 -

  Translocation and subcellular localization of Tir-phiLOV (Movie S1).

  MOV, 846K
